# A toxicokinetic model for thiamethoxam in rats: implications for higher-tier risk assessment

**DOI:** 10.1007/s10646-013-1047-z

**Published:** 2013-02-22

**Authors:** Agnieszka J. Bednarska, Peter Edwards, Richard Sibly, Pernille Thorbek

**Affiliations:** 1 Syngenta, Jealott’s Hill International Research Centre, Bracknell, RG42 6EY UK; 2 School of Biological Sciences, University of Reading, Whiteknights, Reading, RG6 6AJ UK

**Keywords:** Insecticide, Neonicotinoid, Kinetics, Body burden modelling

## Abstract

Risk assessment for mammals is currently based on external exposure measurements, but effects of toxicants are better correlated with the systemically available dose than with the external administered dose. So for risk assessment of pesticides, toxicokinetics should be interpreted in the context of potential exposure in the field taking account of the timescale of exposure and individual patterns of feeding. Internal concentration is the net result of absorption, distribution, metabolism and excretion (ADME). We present a case study for thiamethoxam to show how data from ADME study on rats can be used to parameterize a body burden model which predicts body residue levels after exposures to LD_50_ dose either as a bolus or eaten at different feeding rates. Kinetic parameters were determined in male and female rats after an intravenous and oral administration of ^14^C labelled by fitting one-compartment models to measured pesticide concentrations in blood for each individual separately. The concentration of thiamethoxam in blood over time correlated closely with concentrations in other tissues and so was considered representative of pesticide concentration in the whole body. Body burden model simulations showed that maximum body weight-normalized doses of thiamethoxam were lower if the same external dose was ingested normally than if it was force fed in a single bolus dose. This indicates lower risk to rats through dietary exposure than would be estimated from the bolus LD_50_. The importance of key questions that should be answered before using the body burden approach in risk assessment, data requirements and assumptions made in this study are discussed in detail.

## Introduction

Risk assessment for mammals is currently based on evaluation of the ratio of the daily exposure divided by the oral LD_50_ (typical a bolus dose) for acute effects or NOEL for chronic effects, e.g. reproduction or parental effects. However, exposure to a chemical does not mean that all of the dose will be bioavailable, as toxicokinetics (TK) (e.g. absorption, elimination) strongly influence the received dose of a toxicant, and it is thus internal concentration at target sites that drives the effect. It has long been acknowledged that effects of toxicants are better correlated with systemically available dose than with the external administered dose (e.g. Morgan et al. 1994). Toxicity relationships based on internal tissue concentrations rather than on external exposure concentrations (e.g. concentration in food) are often far less variable among species, among different chemicals that act by similar toxic mechanisms and among different environmental conditions (McElroy et al. 2010). Although it has been recognised recently that TK may be used to refine chemical risk assessments (EC 2007; OECD 2010), and TK are routinely and successfully used in pharmaceutical research, few internal dose data are routinely generated in toxicological studies of pesticides and biocides, and the use of TK in risk assessment for crop protection products is relatively new (Creton et al. 2009). To understand the relationship between the external and internal concentrations of toxicant better, we need toxicokinetic models that translate an external concentration of a toxicant, which can change in time, to an internal concentration at a target site as a function of time. Such models allow for predictions of concentrations of toxicant in the body for different realistic exposure scenarios and enable interspecies extrapolation, which may reduce the need for animal testing.

Internal concentration is the net result of absorption, distribution, metabolism and excretion (ADME), and TK is a mathematical description of these processes. In its simplest form, a one-compartment model with first-order kinetics includes the processes of absorption and elimination, but more complex models may include biotransformation processes or internal distribution (Jager et al. 2011). Multi-compartment models, such as physiologically based pharmacokinetics (PBPK), frequently have many variables and biochemical and physico-chemical determinants (Krishnan and Peyret 2009). Although such complex models that estimate residue levels in specific organs may be sometimes preferred, relatively simple models that track total body burden may be more practical and sufficient for wildlife risk assessment of pesticides where a strong need is felt for relatively simple models that can be applied in complex situations (Fite et al. 2001; Hunka et al. 2012). Nevertheless, the choice of an appropriate TK model depends on the question addressed.

Here we develop a toxicokinetic model for a neonicotinoid, thiamethoxam that can be used to predict internal exposure for a wide range of exposure scenarios including different timescales of exposure and behavioural factors such as feeding pattern in study. As there is a general drive to reduce animal testing, it is desirable if toxicokinetic models can be parameterised based on existing studies. Here we show how common studies such as ADME studies, typically carried out as part of the legal registration requirements (Tomizawa and Casida 2005), can be used and we explore how crucial assumptions of the model can be tested. To illustrate the usefulness of ADME data from an early stage of a study for higher-tier risk assessment, a simple model was developed that considers the absorption of a pesticide across the intestinal wall after oral uptake and its subsequent elimination from the body. To identify the main physiological processes and the level of detail with which organisms have to be described the following questions were considered: (1) can kinetic processes be described as first-order for absorption and elimination of thiamethoxam? (2) how many compartments (tissues or organs) should be included in the model? Is it necessary to represent target organ(s) as separate compartment(s) or is the toxicant concentration in the systemic circulation (blood) sufficient? Because feeding pattern may determine toxicity of chemicals for animals living in natural environment, we also checked (3) how different feeding scenarios influence the internal dose of toxicant in the body? Understanding both physiological and ecological processes will contribute to a better understanding of the risk of different patterns of use of pesticides.

## Materials and methods

All data used in this analysis originate from unpublished GLP studies on the ADME processes of a neonecotinoid, thiamethoxam (Syngenta, *unpubl*.).

### Thiamethoxam

Thiamethoxam is one of the seven neonicotinoid insecticides currently on the market (Jeschke et al. 2010). It is a highly effective systemic and contact insecticide with relatively low mammalian toxicity (Maienfisch et al. 2001). Neonicotinoids are the most important new class of insecticides for integrated pest and insect resistance management programmes (Jeschke and Nauen 2008) that act as agonists of the insect nicotinic acetylocholine receptors (AChRs) (Matsuda et al. 2001). Although neonicotinoids have been extensively studied, ADME studies in mammals have been published only for clothianidin (Yokota et al. 2003).

### Animals

The experiment was performed according to 94/79/EC (Commission Directive 1994), OECD 417 (OECD 1984) and US-EPA FIFRA 85-1 (EPA 1984) guidelines. Laboratory rats (*Rattus norvegicus*) about 7–9 weeks old derived from laboratory culture (CIBA-GEIGY limited, Switzerland) were acclimatized to laboratory conditions for at least 5 days and were separated and individually kept in metabolism cages 1 day before the experiment started. The animals were allowed free access to certified standard diet (Nafag No. 890, NAFAG, Gossau, Switzerland), except the night before administration of ^14^C labelled thiamethoxam. Tap water was offered ad libitum at all times.

### Experimental design

Thiamethoxam (3-(2-chloro-thiazol-5-ylmethyl)-5-methyl-[1,3,5]oxadiazinan-4-ylidene-*N*-nitroamine, CAS 153719-23-4 or CGA 293343 (Syngenta code no) was ^14^C labelled in two positions on the molecule, [Thiazol-2-^14^C] and [Oxadiazin-4-^14^C]. Radiochemical purity was >97 %. Three male and three female rats were randomly assigned to each of the following treatment groups, to receive either a single intravenous (i.v.) dose of 5 mg kg^−1^ body weight (bw), or a single oral (p.o.; Latin *per os*: by mouth) dose of 5 (low dose) or 100 (high dose) mg kg^−1^ bw. For the intravenous administration the test substance was dissolved in 0.9 % NaCl and about 0.3 ml of the solution was intravenously injected via syringe directly into the tail vein. For the oral exposure, test substance was suspended in mixture of polyethylene glycol 200/ethanol 5/3 (v/v) at expected nominal concentrations and each animal received about 0.8 ml of administration solution by stomach tube. Blood samples were collected from three animals of each group. Samples were taken from the tail at 0.25, 0.5, 1, 2, 4, 8, 12, 24, and 48 h after administration.

In addition to the collection of blood, samples of urine and faeces were collected separately from metabolic cages at time intervals of 0–8, 8–24, 24–48, 48–72, 72–96, 96–120, 120–144, 144–168 h after dosing. Additionally, three groups of male and three groups of female rats were used to study tissue residues of thiamethoxam after oral exposure to a low dose of [Thiazol-2-^14^C], a high dose of [Thiazol-2-^14^C] and a low dose of [Oxadiazin-4-^14^C]. The tissues and organs (bone, brain, abdominal fat, testes/ovaries, heart, kidney, liver, lungs, plasma, skeletal muscle, spleen, uterus, whole blood, residual carcass) were sampled by dissection of euthanized animals at four time points as follows: time of maximal concentration of radioactivity (*C*_*max*_) in the blood, time of depletion to ½*C*_*max*_, and 12 and 24 h after thiamethoxam administration. Volumes or weights of each sample were recorded prior to analysis. At each time point, tissue residues were determined in three males and three females after oral administration of [Thiazol-2-^14^C] at both 5 and 100 mg kg^−1^ bw and of [Oxadiazin-4-^14^C] at 5 mg kg^−1^ bw.

The appearance and the behaviour of animals were observed during the course of experiment to safeguard the welfare of the animals. The procedures involving animals were carried out in accordance with a protocol approved by the UK Home Office Animal Care and Use Committee.

### Chemical analysis

Radiopurity was checked by thin layer chromatography (TLC) and high performance liquid chromatography (HPLC) at the time of dosing and shown to be stable. Radioactivity in blood, bone, lungs, gastrointestinal tract, faeces, and carcass was determined by combustion and liquid scintillation counting (LSC). Radioactivity in brain, fat, heart, kidneys, liver, muscle, spleen, gonads, and uterus was determined after digestion with Irgasolve tissue solubiliser by LSC. The results were expressed as μg thiamethoxam equivalents g^−1^ wet tissue or μg thiamethoxam equivalents ml^−1^ wet tissue. All details concerning measurements of radioactivity, TLC, HPLC and calculations performed on experimental data are described in Syngenta report (Syngenta, *unpubl*.). The data were analyzed on the basis of total radioactivity in each studied tissue. The results for blood samples were recalculated based on the relationship that 1 ml of blood is approximately equivalent to 1.06 grams of blood and expressed as μg thiamethoxam ml^−1^.

### Model selection and parameters estimation

Blood concentrations were used to determine kinetics parameters using a commercial software program WinNonlin Version 5.3 (Pharsight Corporation, Mountain view, CA, USA) (see Gabrielsson and Weiner 2000 for more details). Compartmental methods were used and parameters were estimated from the statistical best-fits of the model to experimental time-course data. Weighting of the data using the inverse of the observed plasma concentration (i.e. reciprocal of the observed values) improved the fit of the model and was used in all cases. The model parameters were estimated using the Marquardt method and parameters were checked for significance using asymptotic 95 % confidence intervals.

A one-compartment model was used to calculate toxicokinetic parameters separately for each individual in order to include the variability in TK parameters amongst individuals in statistical analysis of the data. The primary compartmental parameters calculated were *k*_*a*_ (first-order absorption rate constant), *k*_*e*_ (first-order elimination rate constant) and ratio *V/F* where *V* is a volume of distribution (apparent volume which a pesticide distributes into) and *F* is bioavailability, which is determined by absorption across gastrointestinal membranes and hepatic extraction. Degradation of pesticide in gut and fecal excretion also affects *F*. The reason for the ratio *V/F* is due to the inability to determine *F* and *V* separately. This is an inherent limitation of the model and unique values for *F* and *V* can be determined only with information following an intravenous dose.

Area under the zero moment curve (*AUC*) was calculated and used to estimate bioavailability. The relative bioavailability between the two routes of administration, i.e. the fraction of thiamethoxam that was absorbed (unit less fractional bioavailability, *F*) was calculated for each individual separately according to the following equation:

$$ F = {{\left[ {{{AUC_{p.o.} } \mathord{\left/ {\vphantom {{AUC_{p.o.} } {dose_{p.o.} }}} \right. \kern-0pt} {dose_{p.o.} }}} \right]} \mathord{\left/ {\vphantom {{\left[ {{{AUC_{p.o.} } \mathord{\left/ {\vphantom {{AUC_{p.o.} } {dose_{p.o.} }}} \right. \kern-0pt} {dose_{p.o.} }}} \right]} {\left[ {{{AUC_{i.v.} } \mathord{\left/ {\vphantom {{AUC_{i.v.} } {dose_{i.v.} }}} \right. \kern-0pt} {dose_{i.v.} }}} \right]}}} \right. \kern-0pt} {\left[ {{{AUC_{i.v.} } \mathord{\left/ {\vphantom {{AUC_{i.v.} } {dose_{i.v.} }}} \right. \kern-0pt} {dose_{i.v.} }}} \right]}} $$

where *p.o* and *i.v* denote oral and intravenous exposure, respectively. *AUC*_*p.o*._ was calculated for each individual separately and *AUC*_*i.v.*_ was calculated as a mean value for male and female rats separately.

### Statistical analysis of model parameters

A multifactorial ANOVA with body mass as a covariate was used to test differences in absorption rate constant (*k*_*a*_) and bioavailability (*F*) between sexes, labelling position ([Thiazol-2-^14^C] and [Oxadiazin-4-^14^C]) and doses, as well as interactions between factors for oral exposure. If significant differences were concluded among the levels of a factor, then means were separated with LSD tests. Two-way ANOVA with body mass as a covariate and exposure route and sex as explanatory factors was used to check for possible differences in *k*_*e*_ and *V*. If nonsignificant (*p* > 0.05), the covariate was removed from models. A Pearson correlation was used to test for correlations between thiamethoxam concentrations in different tissues. Differences in the regression intercepts and slopes between tissues were tested for their relationship between residues of thiamethoxam and time within the exposure groups using comparison of regression lines. Statistical analyses used the Statgraphics Centurion XV program version 16.1.11.

### Body burden model

#### Body burden model description

The values for absorption and elimination rate constants estimated from fitting one-compartment model to the radiolabelled data (WinNonlin analysis) were used to simulate the change of the pesticide body weight-normalized dose in the body with time for different feeding scenarios. For this purpose, the internal tissues of the organism excluding the gastro-intestinal tract (the content of which is not strictly ‘in’ the organism) were treated as a single compartment. Thus the animal ingests food with residues of a toxicant, the toxicant is absorbed from the gastro-intestinal tract into the bloodstream and transported to target organ(s), and then is eliminated from the body. Elimination may occur by several routes including loss in urine and faeces. The rates of change in the doses of thiamethoxam in the gut and bloodstream were described mathematically as the difference between compartment rates of uptake and loss. Exchange rates between compartments represent physical transfers of a substance, as biotransformation of thiamethoxam to metabolites was not taken into account in the model. No distinction was made between the rate of the loss of pesticide from the gastrointestinal tract and its appearance in the systemic circulation; what is lost from the gastrointestinal tract all appears in the systemic circulation each time unit.

#### Body burden model implementation

In order to simulate the change of the pesticide dose in the gut and in the body with time the following equations were implemented in an Excel spreadsheet:

$$ \Updelta D_{gut} = I - k_{a} D_{gut} F $$

$$ \Updelta D_{\text{int}} = k_{a} D_{gut} F - k_{e} D_{\text{int}} $$

where $\Updelta D$ indicates change in the body weight-normalized dose of pesticide in given time interval, here one minute; subscripts *gut* and *int* denote gut and internal (bloodstream), respectively; *I* indicates ingestion rate (i.e. the rate of toxicant transfer from exposure dose to the gut, mg a.i. kg^−1^ bw min^−1^); *F* represents bioavailability, here *F* = 1 (see Results); $k_{a}$ represents the rate of toxicant absorption from the gut into the system (min^−1^), and $k_{e}$—the rate of toxicant elimination from the system (min^−1^).

#### Body burden model verification

To verify the body burden model was performing in a reasonable manner (i.e. that implementation was correct) and could be used regardless of exposure levels (even though difference in *k*_*a*_ between doses was found), we ran simulations representing both low- and high-level of exposure (0.5 and 100 mg a.i. kg^−1^ bw, respectively) with different combinations of *k*_*a*_ and *k*_*e*_. The pesticide movement to the gut and bloodstream was monitored and the predicted shapes of the curve were visually compared with measured data to check that the model reproduced results correctly.

#### Simulation of thiamethoxam doses in the body at different feeding scenarios

Different scenarios of exposure were tested to check effect of feeding pattern on the change of thiamethoxam dose both in the gut and in the system as a function of time: (1) LD_50_ given as a bolus dose (i.e. all dose eaten during 1 min); (2) LD_50_ dose eaten with constant ingestion rate of 13 mg a.i. kg^−1^ bw min^−1^ (i.e. all dose eaten within 2 h); (3) LD_50_ dose eaten with constant ingestion rate of 6.5 mg a.i. kg^−1^ bw min^−1^ (i.e. all dose eaten within 4 h); (4) LD_50_ dose eaten with constant ingestion rate of 13 mg a.i. kg^−1^ bw min^−1^ within 2 h in total but with 4 h break after the first hour of feeding. All simulations were run with high mean *k*_*a*_ and low mean *k*_*e*_ rate constants (worst-case). The acute oral LD_50_ value calculated after bolus gavage exposure of rats was 1563 mg kg^−1^ (Maienfisch et al. 2001; EPA 2002). The maximum internal doses (max *D*_*int*_) were used as a metric for comparison between different exposure scenarios.

## Results

Thiamethoxam was rapidly perfused throughout the body and rapidly eliminated: the levels of ^14^C in measured tissues were close to or less than limit of detection/quantification 24 h after administration of 0.5 mg kg^−1^ bw. The results indicated no accumulation in any of the tissues examined. The residues of thiamethoxam in blood and other tissues were highly correlated (*r* ≥ 0.9, *p* ≤ 0.0001 for all studied tissues) suggesting that tissues rapidly reach and maintain equilibrium with blood. Therefore, the kinetics parameters for blood could be used for other tissues for prediction of internal dose after exposure to LD_50_ dose. Moreover, overall elimination from all tissues was similar and fast and in all exposure groups except two the differences were found only in intercepts (*p*_*intercepts*_ < 0.0001, *p*_*model*_ < 0.0001, *r* ^*2*^ ≥ 88.5 %). The only two cases in which significant differences among the slopes were found (*p* ≤ 0.0001) were females exposed to 100 mg [Thiazol-2-^14^C] kg^−1^ bw (elimination of thiamethoxam from heart, bone and kidneys was faster than in other studied tissues), and males exposed to 0.5 mg [Oxadiazin-4-^14^C] kg^−1^ bw (slower elimination of thiamethoxam from brain, abdominal fat and liver in comparison with other tissues).

### Model selection and estimation of parameter values

**Fig. 1:**
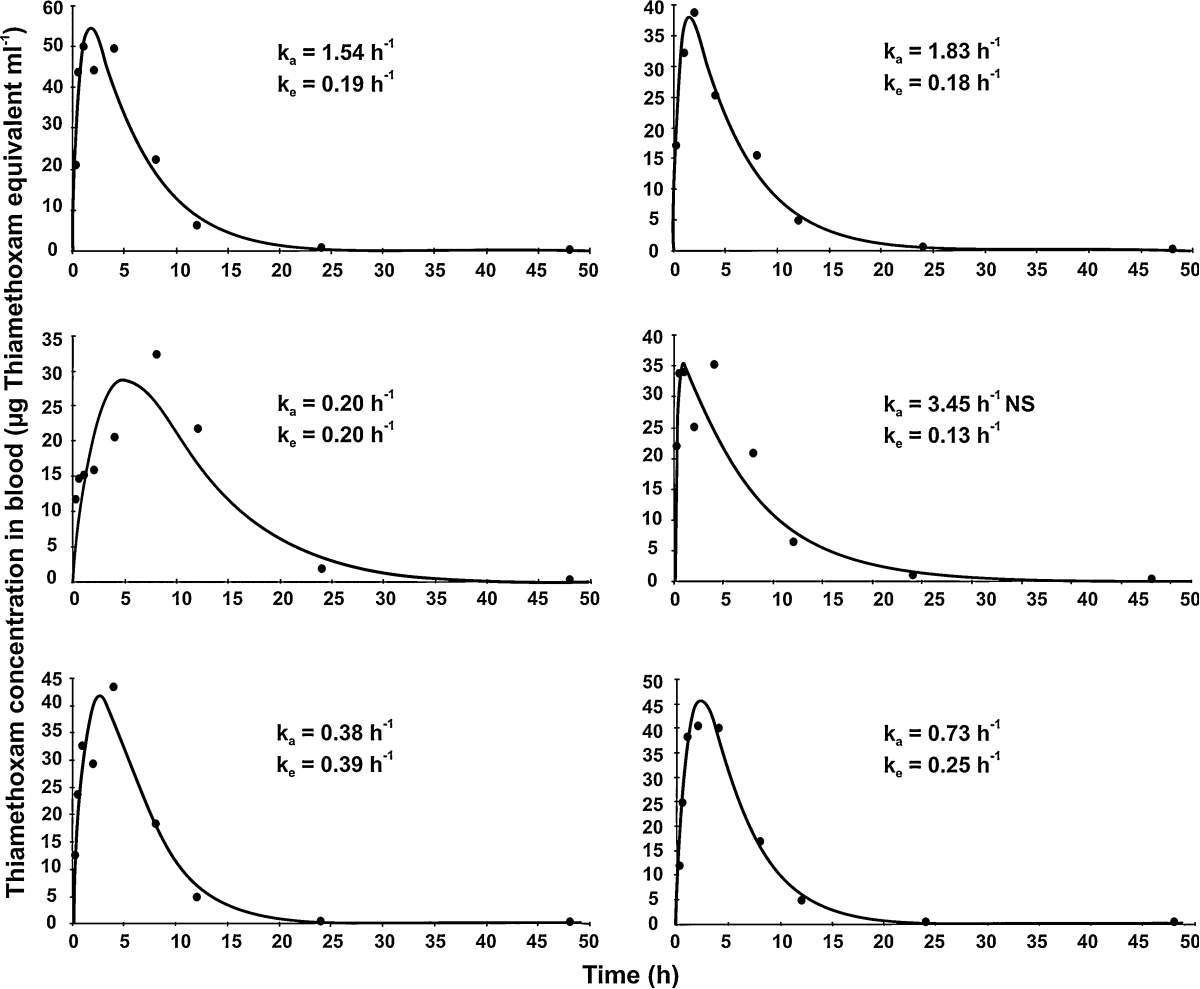
Concentration of thiamethoxam in the blood of three male rats (*left-hand column*) and three female rats (*right-hand column*) administered 100 mg kg^−1^ bw [Oxadiazin-4-^14^C]. Lines are the compartmental toxicokinetic model fits to the experimental blood data. Note different scales on *y* axis; *NS* non-significant

**Fig. 2:**
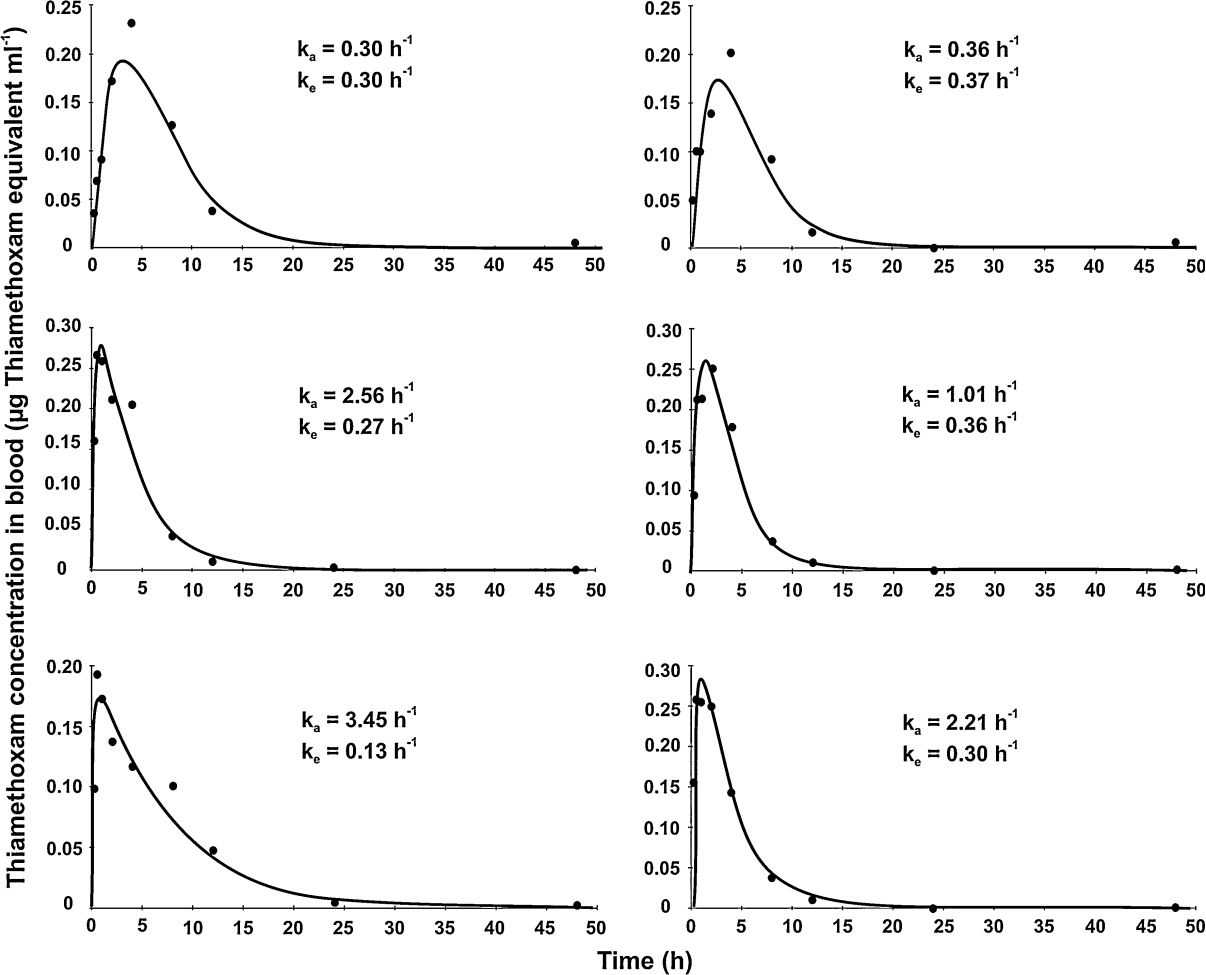
Concentration of thiamethoxam in the blood of three male rats administered 0.5 mg kg^−1^ bw [Thiazol-2-^14^C] (*left-hand column*) or 0.5 mg kg^−1^ bw [Oxadiazin-4-^14^C] (*right-hand column*). Lines are the compartmental toxicokinetic model fits to the experimental blood data. Note different scales on *y* axis

Compartmental analysis was used to determine values of kinetic parameters for further modelling (Table 1). The one-compartment first-order model gave the best fit to the data based on visual examination of the fitted curves, residual plots, and Akaike’s information criterion (AIC), regardless of the dose (0.5 or 100 mg a.i. kg^−1^ bw), exposure route (oral or intravenous) or sex. Fits of the one-compartment model are shown in Figs. 1, 2. Variability among individuals in toxicokinetic parameters was observed irrespective of dose and exposure route, so likely reflects natural variation among individuals. *k*_*a*_ was higher at 0.5 than 100 mg a.i. kg^−1^ bw (*p* = 0.014) using body mass as a covariate (*p* = 0.03). As body weight was confounded with sex (females were larger, *p* < 0.003), it was not possible to distinguish between them. Therefore, body mass and sex (continuous variable) was included in the general linear model as the interaction term. The effect of interaction between sex and body mass on *k*_*a*_ was not significant (*p* > 0.7). None of studied variables affected *k*_*e*_ in orally exposed rats, but *k*_*e*_ was higher in rats exposed to 0.5 mg kg^−1^ bw [Thiazol-2-^14^C] intravenously than orally (*p* = 0.05). Sex did not affect *AUC*.

**Table 1:** Toxicokinetic parameters for 0.5 mg Thiazol-2-^14^C kg^−1^ bw (intravenous exposure) and 0.5 and 100 mg Thiazol-2-^14^C and Oxadiazin-4-^14^C kg^−1^ bw (oral exposure) in rats; mean ± SD (upper row) as well as minimum and maximum values (lower row) for each group are indicated

Parameters	Intravenous exposure		Oral exposure							
	[Thiazol-2-^14^C] 0.5 mg kg^−1^ bw		[Thiazol-2-^14^C] 0.5 mg kg^−1^ bw		[Oxadiazin-4-^14^C] 0.5 mg kg^−1^ bw		[Thiazol-2-^14^C] 100 mg kg^−1^ bw		[Oxadiazin-4-^14^C] 100 mg kg^−1^ bw	
	Male	Female	Male	Female	Male	Female	Male	Female	Male	Female
k_a_ (h^−1^)	–	–	2.1 ± 1.62	2.3 ± 1.93	1.20 ± 0.93	3.2 ± 0.29	0.78 ± 0.32	1.6 ± 0.71	0.71 ± 0.73	2.00 ± 1.37^a^
			0.30–3.45	0.38–4.23	0.37–2.20	2.9–3.49	0.41–0.98	0.87–2.3	0.20–1.54	0.72–3.45
k_e_ (h^−1^)	0.26 ± 0.02	0.50 ± 0.11	0.23 ± 0.09	0.23 ± 0.13	0.34 ± 0.034	0.25 ± 0.06	0.28 ± 0.12	0.18 ± 0.06	0.25 ± 0.11	0.19 ± 0.06
	0.24–0.28	0.39–0.60	0.13–0.30	0.13–0.38	0.30–0.37	0.18–0.30	0.19–0.41	0.12–0.24	0.19–0.39	0.13–0.25
Absorption half-life (min)^b^			19.8	18.1	34.7	13.0	53.3	26.0	38.6	20.8
Elimination half-life (min)	160.0	83.2	180.8	180.8	122.3	166.4	148.5	231.0	166.3	218.9
*AUC* (h ug^−1^ ml^−1^)	2.30 ± 0.19	1.63 ± 0.38	1.49 ± 0.15	1.56 ± 0.53	1.30 ± 0.015	1.03 ± 0.13	342 ± 80	278 ± 59	359 ± 50	294 ± 24
F	–	–	0.62 ± 0.11	0.94 ± 0.27	0.67 ± 0.01	0.75 ± 0.12	0.84 ± 0.21	0.94 ± 0.22	0.80 ± 0.11	0.96 ± 0.11

*k*_*a*_ absorption rate constant; *k*_*e*_ elimination rate constant; *AUC* area under the curve; *F* bioavailability

^a^One non-significant *k*_*a*_ value estimated for a female rat dosed at 100 mg [Oxadiazin-4-^14^C] kg^−1^ bw was included in statistical analysis

^b^Half-life = ln(2)/*k*, where *k* is either *k*_*a*_ or *k*_*e*_ for absorption and elimination half-lives, respectively; only mean values are shown

The relative bioavailability calculated from *AUC* after dose-normalization ranged from 0.62 to 0.96 (Table 1) and was significantly lower in males than females (*p* = 0.02) as well as at 0.5 than 100 mg a.i. kg^−1^ bw (*p* = 0.04). Although the bioavailability determined by the *AUC* ratio after oral and i.v. administration was below 1, complete absorption of thiamethoxam was assumed in body burden modelling (*F* = 1), as the samples of urine and faeces collected separately from metabolic cages indicated that most of the radiolabel (95 %) was excreted via kidneys and only 4 % was found in the faeces. Moreover, the amount eliminated with the faeces was derived from biliary excretion, thus proving complete absorption (data not shown). Therefore the worst-case bioavailability assumption (*F* = 1) was used for further simulations of thiamethoxam dose in the body at different feeding scenarios. None of studied variables affected *V*, and only body mass as a covariate was significant in the model (*p* = 0.04).

### Body burden model

#### Body burden model verification

**Fig. 3:**
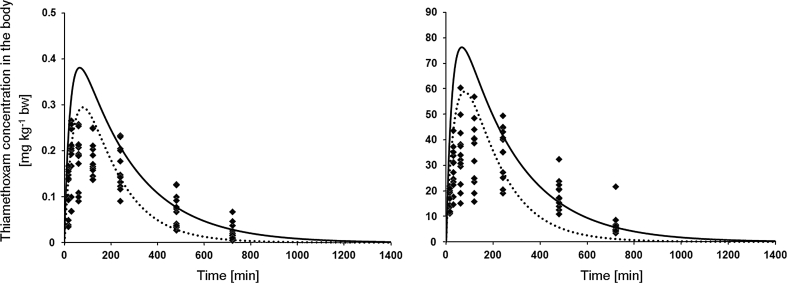
Comparison of body burden levels of thiamethoxam predicted by the model with measured data for rats exposed to 0.5 mg kg^−1^ bw (*left-hand graph*) and 100 mg kg^−1^ bw (*right-hand graph*) showed for two extreme combination of *k*_*a*_ and *k*_*e*_: *k*_*a*_ = 2.2 and *k*_*e*_ = 0.25 (*solid line*) and *k*_*a*_ = 1.3 and *k*_*e*_ = 0.4 (*dotted line*)

Because *k*_*a*_ and *k*_*e*_ varied with dose and exposure route, respectively, we examined the effects of different combinations of *k*_*a*_ and *k*_*e*_ on body burden to see how important this is for risk. Therefore, means (±SD) for *k*_*a*_ were calculated separately for rats exposed to low and high doses (2.2 ± 1.37 and 1.3 ± 0.94, respectively) and means for *k*_*e*_ were calculated for i.v. and orally exposed rats (0.4 ± 0.15 and 0.25 ± 0.09, respectively). The predicted internal dose–time curves for the thiamethoxam levels in the body at two extreme combinations of *k*_*a*_ and *k*_*e*_ (i.e. high mean *k*_*a*_ and low mean *k*_*e*_ or low mean *k*_*a*_ and high mean *k*_*e*_) are shown on Fig. 3. The model exhibited the expected general patterns with regard to thiamethoxam movement to the gut and bloodstream regardless of which combination of parameters was used. There was substantial variability between individuals, but the time at which the peaks were reached, as well as the shapes of the internal dose–time curve were reflected correctly. Although for high mean *k*_*a*_ and low mean *k*_*e*_ the predicted peaks were higher than the measurements (probably because the model assumed *F* = 1 for all individuals), this combination of kinetics parameters were used for further simulations as the most protective, i.e. conservative approach (Fig. 3).

#### Simulation of thiamethoxam doses in the body at different feeding scenarios

**Fig. 4:**
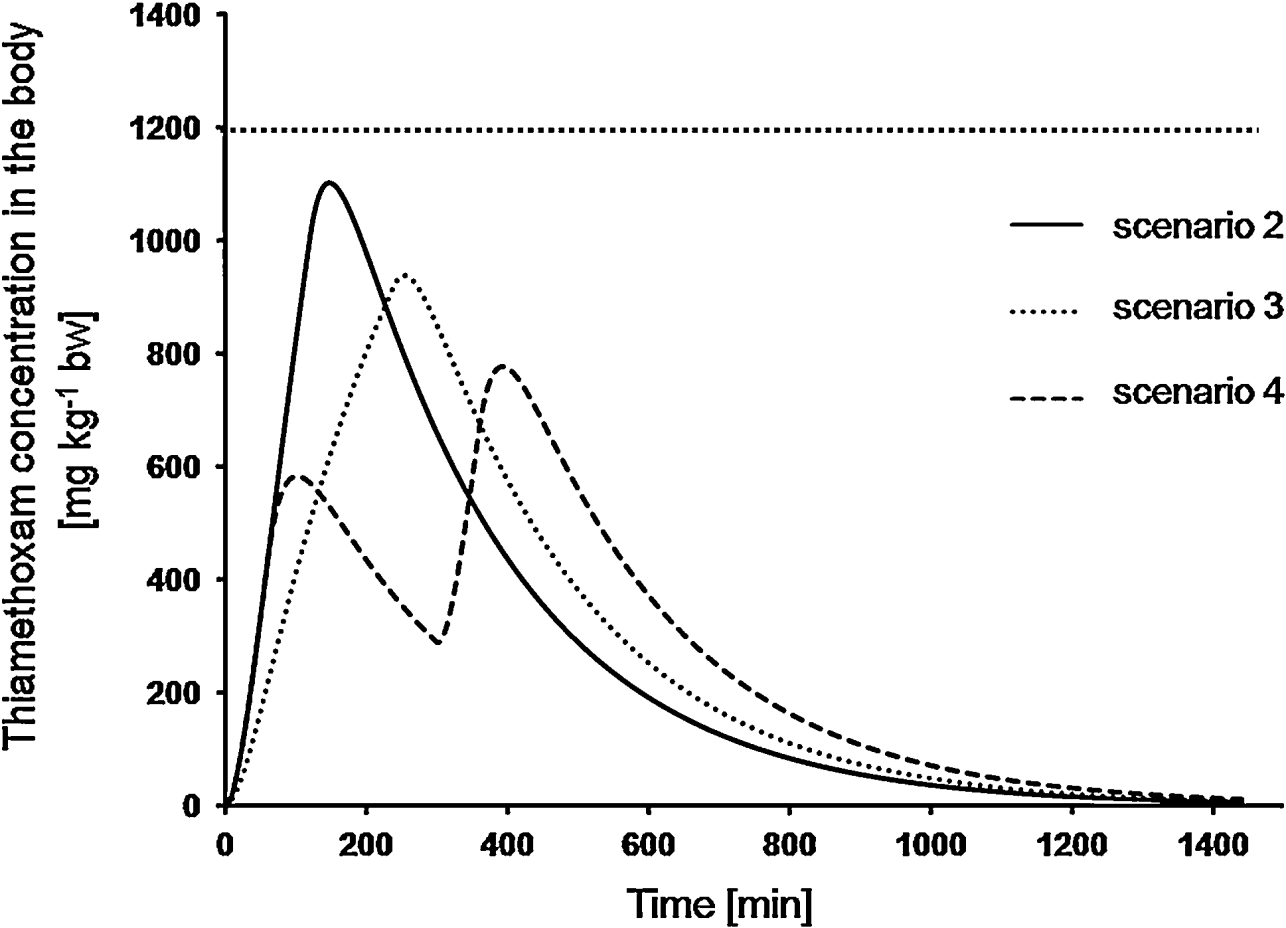
Body burden (i.e., body weight-normalized internal dose) levels of thiamethoxam predicted by the model for rats exposed to LD_50_ according to different feeding scenarios (see Materials and methods for scenarios description); *vertical dotted line* indicates maximum internal dose of thiamethoxam in the body after bolus gavage exposure to LD_50_; all simulations run for *k*_*a*_ = 2.2 and *k*_*e*_ = 0.25

According to our expectations, the highest max *D*_*int*_ were reached when the LD_50_ was given as a bolus dose (1193 mg kg^−1^ bw). In the two feeding scenarios with continuous feeding, maximum doses of thiamethoxam in the body were: 1102 and 939 mg kg^−1^ bw, for scenario 2 and 3, respectively. The lowest max *D*_*int*_ was obtained in the scenario when LD_50_ was eaten in two one-hour feeding bouts separated by a four-hour non-feeding break (777 mg kg^−1^ bw) (Fig. 4).

## Discussion

We developed a simple toxicokinetic model to predict internal doses of thiamethoxam in rats after oral exposure. The model was parameterised using data from ADME studies, often available for rats, live-stock or hen (EFSA 2009). The advantages of using the ADME data in study design and dose selection to avoid the use of unrealistic doses has been already extensively discussed as a refinement option to reduce animal testing (Barton et al. 2006; Creton et al. 2009). The present work focuses on integration of ADME study in higher-tier risk assessment, thus extending the use of existing data, and provides a framework for simple TK modelling without complex details of the physiological mechanisms, yet it captures the dynamics of the internal doses of thiamethoxam. Using the simple model we showed that different feeding patterns may influence internal dose of thiamethoxam. However, before using simple models for other chemicals it is important to understand what data are needed to check the assumptions and the advantages and limitations of such approach, as well as where and how it should be adapted or extended.

The requirements for our simple body burden model are data on concentration of toxicant in blood (or other tissue) over time to estimate absorption and elimination rate constants and it should be checked if kinetics are first-order or more complex. Moreover, if urine and faeces samples are not available, the kinetics data for i.v. and oral bolus doses can give rough estimates of the bioavailability (F) of the pesticide. The conventional way of estimating bioavailability is by sequential administration of the systemic (i.v.) and extravascular (here oral) doses with an interval of one day (or even a week) between administration and with an underlying assumption that the clearance is constant between the two administrations. However, some chemicals can induce (or inhibit) enzymes and thus, affect the time course of the second dose (Gabrielsson and Weiner 2000). The values obtained for *F* from analysis on different treatment groups, together with information that only 4 % of the radiolabel was found in the faeces, proved high bioavailability. Therefore, the worst-case assumption in the case of bioavailability (*F* = 1) was adopted in this study. It is also the most conservative approach from a risk assessment point of view.

While *k*_*a*_ and *k*_*e*_ are key parameters, the distribution of the chemical in tissues is important to define the target organ(s), and metabolism where the molecule is rapidly transformed. It can also provide information on the potential of a test substance and its metabolites for accumulation and persistence in some tissues. The compartmental analysis gives an indication of how many compartments are needed, but it is important to identify the target organ, and site of chemical metabolism. Our study indicated no accumulation of thiamethoxam in any tissues measured, including fat, which can be an important compartment for lipophilic compounds (Van Eijkeren et al. 2006). Because the concentration of thiamethoxam in blood was highly correlated with the concentrations in other tissues, the blood could be used as a single compartment. This compartment represents all tissues (i.e. the whole body burden excluding the digestive tract) in which an internal concentration reaches equilibrium with the concentration in blood within a few hours. However if target tissue/organ(s) concentrations are poorly correlated with blood concentration, such simple models may produce unreliable predictions, as toxicological responses may be a function of residue levels in specific tissues. Moreover, if metabolites significantly influence the overall toxicity of a chemical, the more elaborate analysis of TK may be required.

One important limitation from this type of ADME study is that all measurements were based on total ^14^C radioactivity as a surrogate for the test substance, meaning that the fractions of parental thiamethoxam and its metabolites were not characterized separately. Therefore both the parent chemical and its metabolites contributed to the reported tissue concentrations. In general, without quantification of the parent compound, the data are unsatisfactorily nonspecific (Barton et al. 2006), as such data may not be representative of the kinetics of the relevant metabolite at the target site (Rubach et al. 2011). Fischer (2005) recently suggested that for modern pesticides, that generally do not bioaccumulate, a TK model capable of realistically modelling metabolic processes and the site of toxic action needs to be developed. However, after oral dosing of rats, up to 90 % of the applied thiamethoxam at 100 mg kg^−1^ bw is readily eliminated as parent compound in the urine (Maienfisch et al. 2001). It may be assumed with rapid excretion that exposure to biotransformation enzymes is limited. Therefore, the metabolizing tissue (liver) was not characterized as a separate compartment in our model. For highly metabolised pesticides, the more elaborate analysis should be linked to the metabolic organisation of the organism and more complex models (e.g. PBPK) can be useful (Krishnan and Peyret 2009). Moreover, different species may respond differently: after systemic administration of thiamethoxam (20 mg kg^−1^) in mice at least 44 % of this pesticide was metabolised (Ford and Casida 2006).

Under field conditions, animals are not likely to eat all their dietary requirements in one bite (i.e. as a bolus dose) but rather via ingestion with a slower feeding rate over much longer periods. Because we showed that higher maximum internal doses were reached when LD_50_ dose was given as an oral gavage bolus (standard dosage in LD_50_ test on mammals, e.g. OECD 2001), it can be expected that the use of gavage dosing results in high systemic levels that induce more adverse effects than if an equivalent dose is given via the diet at slower feeding rates. Here, we propose the use of maximum internal dose (max *D*_*int*_) to estimate risk, as for compounds that are excreted rapidly (such as thiamethoxam) acute effects are usually associated with peaks (Barton et al. 2006). However, for other modes of action, the cumulative exposure (*AUC*) may be a more valid endpoint for comparison between different exposure scenarios. TK is only the first part of risk estimation, as toxicodynamics also affects risk. Recovery is not necessarily immediate; effects can be additive over time at a constant internal dose and might not disappear once the toxicant leaves the system. Therefore, the body burden model should be interpreted with care for different pesticides especially with very different physico-chemical properties in relation to what is known about a toxicant’s mode of action in the species of interest.

When laboratory data are extrapolated to field situations, it is important to know what rates of feeding occur in the field and how they vary. This can be difficult to measure in practice. Animals in the wild are often under pressure to feed fast in order to compete effectively for food, and/or to minimise the time of being exposed to predators. Therefore, the feeding rates achieved in laboratory study (or assumed in the model) have to correspond to maximum rates occurring in the field (EFSA 2009). Methods already exist for estimating food intake rate based on allometric equations for daily energy expenditure of wild eurythermal animals combined with energy and moisture contents and assimilation efficiencies for different foods (Crocker 2005). If information about feeding habit of studied species is not available, hypothetical scenarios may be tested for realistic feeding patterns. The constant uptake of toxicant per unit time was used in all presented scenarios, but testing scenarios with varying ingestion rates would also be possible. Moreover, probabilistic approach allowing to incorporate the full range of values for ingestion rates, and to quantify impacts of variability and uncertainty on risk seems to be good option for real field situations. Similarly, the possible way to include high variability in kinetics parameters between individuals can be by replacing worst-case combination of single fixed values for *k*_*a*_ and *k*_*e*_ with their distributions and cover the full range of outputs.

One of the major challenges for birds and mammals is long term risk assessment, where endpoints estimated from long-term laboratory studies carried out under constant exposure have to match field exposures, where both concentrations in the food and the amount of the food eaten may vary substantially both temporally and spatially.

TK provides additional information if there is strong variation of exposure and/or if internal exposure changes slower than external (rates of TK processes are limiting). Therefore TK is especially relevant for birds and mammals usually exposed by uptake of contaminated food only during feeding times, so usually in the range of a few hours. If long-term exposure needs to be tested, growth of an animal may also need to be taken into account together with the chemical’s half-life; and the amount of pesticide incorporated naturally into food may differ from what is observed in gavage dosing (Smith et al. 2009). Moreover, some animals have developed mechanisms that enable them to avoid contaminated food. Although it is hard to determine precise mechanisms for any given pesticide, avoidance is commonly seen in dietary studies and has the potential to reduce exposure, and hence risk, in the field as it prevents body burdens from reaching harmful thresholds (EFSA 2009; Thompson 2007).

## Conclusion

Simple one-compartment model with first-order kinetics can be used to predict the internal dose of thiamethoxam in small mammals for the purposes of risk assessments. Our results indicate that rats exposed to thiamethoxam via diet will have lower maximum body burden than those exposed via oral gavage, and the slower they eat the lower the systemic exposure. The model may be re-parameterized for further mammal and avian risk assessment of different chemicals and used to describe TK for other chemicals and for a range of feeding rates that cover animals’ feeding behaviour in the field. We have outlined some critical assumptions that need to be checked before developing such models for other chemicals, and made suggestions to how the assumptions may be checked. We conclude that toxicokinetic models are promising for wildlife risk assessments, but good understanding of feeding patterns is needed for accurate estimation of chronic risk.
